# The Registry for Migraine (REFORM) study: methodology, demographics, and baseline clinical characteristics

**DOI:** 10.1186/s10194-023-01604-2

**Published:** 2023-06-12

**Authors:** William Kristian Karlsson, Håkan Ashina, Christopher Kjær Cullum, Rune Häckert Christensen, Haidar Muhsen Al-Khazali, Faisal Mohammad Amin, Messoud Ashina, on behalf of the REFORM Investigators, Afrim Iljazi, Andreas Vinther Thomsen, Basit Ali Chaudhry, Betel Tesfay, Janu Thuraiaiyah, Lili Kokoti, Nadja Bredo Rasmussen, Rogelio Domínguez-Moreno, Thien Phu Do, Zixuan Alice Zhuang

**Affiliations:** 1 Department of Neurology, Danish Headache Center, Copenhagen University Hospital – Rigshospitalet, Valdemar Hansens Vej 5, Glostrup, 2600 Copenhagen, Denmark; 2 Department of Clinical Medicine, Faculty of Health and Medical Sciences, University of Copenhagen, Copenhagen, Denmark; 3 Department of Brain and Spinal Cord Injury, Copenhagen University Hospital – Rigshospitalet, Copenhagen, Denmark; 4 Department of Anesthesia, Critical Care and Pain Medicine, Beth Israel Deaconess Medical Center, Boston, MA USA; 5 Harvard Medical School, Boston, MA USA

**Keywords:** Biomarker, CGRP, Erenumab, Magnetic resonance imaging, Biochemistry, Predictors, Provocation

## Abstract

**Background:**

Erenumab has demonstrated effectiveness for prevention of migraine attacks, but the treatment is costly, and a considerable proportion of patients do not respond to it. The Registry for Migraine study (REFORM) was initiated to discover biomarkers that can predict response to erenumab in patients with migraine. The specific objective was to investigate differences in erenumab efficacy based on clinical information, blood-based biomarkers, structural and functional magnetic resonance imaging (MRI), and response to intravenous infusion of calcitonin gene-related peptide (CGRP). In this first report of the REFORM study, we provide a comprehensive description of the study methodology, and present the baseline characteristics of the study population.

**Methods:**

The REFORM study was a single-center, prospective, longitudinal cohort study in adults with migraine who were scheduled to receive preventive treatment with erenumab as part of a separate, open-label, single-arm phase IV trial. The study included four periods: a 2-week screening period (Weeks -6 to -5), 4-week baseline period (Week -4 to Day 1), 24-week treatment period (Day 1 to Week 24), and a 24-week follow-up period without treatment (Week 25 to Week 48). Demographic and clinical characteristics were recorded using a semi-structured interview, whilst outcome data were obtained using a headache diary, patient-reported outcomes, blood sampling, brain MRI, and responsiveness to intravenous infusion of CGRP.

**Results:**

The study enrolled 751 participants, with a mean age ± SD of 43.8 ± 12.2 years, of which 88.8% (*n* = 667) were female. At enrollment, 64.7% (*n* = 486) were diagnosed with chronic migraine, and 30.2% (*n* = 227) had history of aura. The mean monthly migraine days (MMDs) was 14.5 ± 7.0. Concomitant preventive medications were used by 48.5% (*n* = 364) of the participants, and 39.9% (*n* = 300) had failed ≥ 4 preventive medications.

**Conclusion:**

The REFORM study enrolled a population with a high migraine burden and frequent use of concomitant medications. The baseline characteristics were representative of patients with migraine in specialized headache clinics. Future publications will report the results of the investigations presented in this article.

**Trial registration:**

The study and sub-studies were registered on ClinicalTrials.gov (NCT04592952; NCT04603976; and NCT04674020).

**Supplementary Information:**

The online version contains supplementary material available at 10.1186/s10194-023-01604-2.

## Introduction

The advent of drugs targeting calcitonin gene-related peptide (CGRP) signaling has expanded the therapeutic armamentarium for migraine [1]. Erenumab, the first drug of this class, was approved in 2018 and has been shown to be effective and well-tolerated for the preventive treatment of both episodic and chronic migraine [1–3]. However, a considerable proportion of people with migraine do not respond to treatment with erenumab [4, 5], and its availability is currently limited by high costs and strict reimbursement policies [6]. Reliable predictors of erenumab efficacy are thus needed for achieving precision medicine and optimal resource allocation.

Predictive biomarkers represent a promising approach, in which a candidate biomarker is measured in a patient before treatment, and can, if proven valid, be used to predict the response to a particular drug [7, 8]. In recent years, studies have investigated candidate biomarkers for prediction of erenumab efficacy in patients with migraine, using CGRP measurements in blood or saliva [9–11] and magnetic resonance imaging (MRI) [12, 13]. These studies have provided some insights but were limited by methodological issues and small samples [9–13].

The present Registry for Migraine (REFORM) study is a large, single-center, prospective, longitudinal cohort study in adults with migraine. The study comprises four cores: Clinical Core, Biochemistry Core, MRI Core, and Provocation Core. The overall aim is to investigate differences in erenumab efficacy based on clinical data, blood-based markers, structural and functional MRI markers, and responsiveness to intravenous infusion of CGRP. Differences in blood-based markers and MRI outcomes will also be compared between participants with migraine and healthy controls. In this initial report of the REFORM study, we describe the design and methodology, and present the baseline characteristics of the study population.

## Methods

### Study oversight and reporting

REFORM was approved by the relevant ethics committee and the Danish Data Protection Agency. The study was conducted following the principles of the Declaration of Helsinki [14], and all participants provided written informed consent before undergoing any study-related procedures. The study was reported in accordance with the Strengthening the Reporting of Observational Studies (STROBE) statement [15].

### Study design

REFORM, a single-center, prospective, longitudinal cohort study, was conducted at the Danish Headache Center; a specialized tertiary care center for headache disorders. The study and its sub-studies have been registered on ClinicalTrials.gov (NCT04592952; NCT04603976; and NCT04674020).

### Participants

We contacted patients of the Danish Headache Center, who were scheduled to receive 140 mg subcutaneous erenumab every fourth week (28^th^ day) for a total of 24 weeks as part of a separate open-label, single-arm, phase IV trial (NCT04265755).

Eligible participants were ≥ 18 years of age and had a ≥ 1 year history of migraine in accordance with the 3^rd^ edition of the International Classification of Headache Disorders (ICHD-3) [16]. Participants were required to report ≥ 4 monthly migraine days (MMDs) on average across the past three months prior to screening. Co-existing medication-overuse headache (MOH) diagnosed according to ICHD-3 was permitted. Preventive migraine medications, including onabotulinumtoxinA, were also permitted provided stable dosage ≥ 2 months prior to screening and throughout the study.

Key exclusion criteria were onset of migraine ≥ 50 years of age, and history of hemiplegic migraine, cluster headache, or any ongoing secondary headache disorder other than MOH. Participants were also excluded if they had previously received treatment with erenumab, or any other monoclonal antibody (mAb) against CGRP signaling within three months of screening. No specific exclusion criteria were imposed regarding the use of small molecule CGRP receptor antagonists, as these drugs had not obtained approval for the treatment of migraine at the time of study commencement. The complete list of inclusion and exclusion criteria for each REFORM Core is provided in Supplementary Appendix 1 (Clinical and Biochemistry Cores) and Supplementary Appendix 2 (MRI and Provocation Cores).

### Healthy controls

A control group of healthy individuals were also enrolled. These participants were matched on age, sex, and age-distribution with participants in the MRI Core. Controls were required not to have any ongoing or history of clinically significant medical conditions, any psychiatric disorders, or headache disorders except for infrequent episodic tension-type headache. We also excluded individuals who had a family history of primary headache disorder, other than ≤ 5 days per month of tension-type headache. The complete list of inclusion and exclusion criteria for healthy controls is provided in Supplementary Appendix 3.

### Procedures

**Fig. 1:**
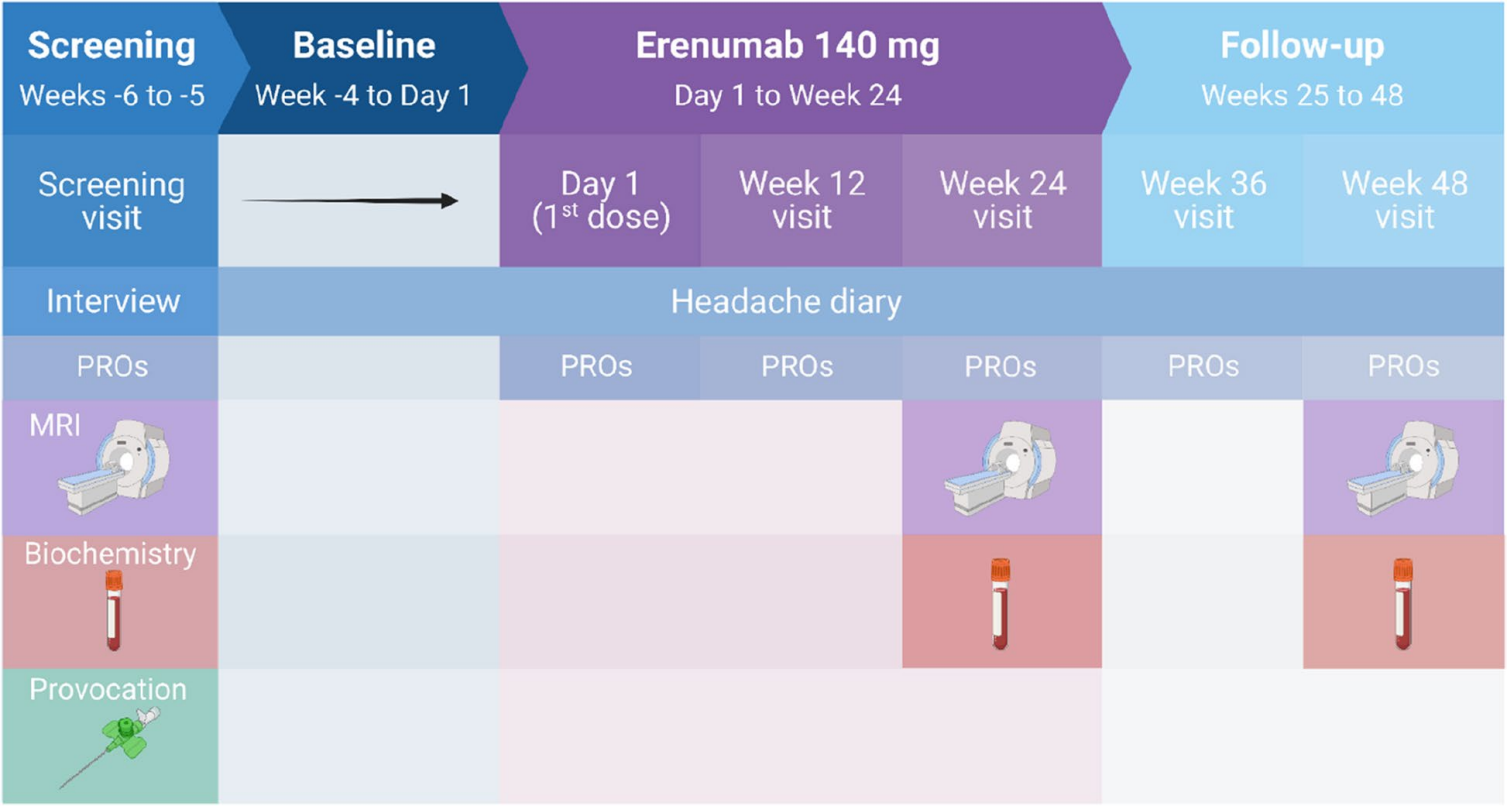
Study overview. All participants were enrolled in the REFORM Clinical Core, whilst participation in the Biochemistry, MRI, and Provocation Core was optional. The Clinical Core consisted of four periods: a 2-week screening period (Weeks -6 to -5), a 4-week baseline period (Week -4 to Day 1), a 24-week treatment period (Day 1 to Week 24), and a 24-week follow-up period without treatment (Weeks 25 to 48). Participants were evaluated for study eligibility and underwent a semi-structured interview at the Screening visit (Weeks -6 to -5). Those who participated in the Biochemistry and/or MRI Cores had their blood samples collected at three different timepoints: Screening (Weeks -6 to -5), Week 24 (± 2 weeks), and Week 48 (± 4 weeks). For participants who were also involved in the MRI or Provocation Core, blood samples were obtained on the day of their first MRI scan or the day of the CGRP-provocation experiment. Participants were asked to complete nine patient-reported outcomes at Screening, Day 1 (1^st^ dose), and every 12 weeks throughout the treatment period and the subsequent follow-up period without treatment. Healthy controls underwent blood sample collection and MRI scans at one scheduled site visit. Figure was created using BioRender.com

All participants were enrolled in the REFORM Clinical Core, whilst participation in the Biochemistry, MRI, and Provocation Core was optional. An overview of each core is shown in Fig. 1. The Clinical Core included four periods: a 2-week screening period (Weeks -6 to -5), a 4-week baseline period (Week -4 to Day 1), a 24-week treatment period (Day 1 to Week 24), and a 24-week follow-up period without treatment (Week 25 to Week 48). The study had five in-person site visits and one phone visit, which were scheduled as follows: Screening (Weeks -6 to -5), Day 1 (1^st^ dose of erenumab), Week 12, Week 24 (± 2 weeks; end of treatment), Week 36 (phone visit), and Week 48 (± 4 weeks; end of study).

During the 4-week baseline period, participants had to fill out the headache diary on at least 21 of 28 days to receive the first dose of erenumab. Participants who discontinued erenumab during the treatment period, or commenced any mAbs targeting CGRP signaling during the follow-up period, were withdrawn from the study. In cases where participants experienced side effects, the investigator was allowed to reduce the dosage from 140 to 70 mg.

### Clinical core

An overview of the Clinical Core instruments and assessed domains is presented in Supplementary Appendix 4.

#### Screening and semi-structured interview

The screening visit included an assessment of study eligibility, including physical and neurological examination. A semi-structured interview was also conducted to gather information on sociodemographics, medical history, clinical characteristics, and medicines history. Details of the semi-structured interview are presented in Supplementary Appendix 5.

#### Headache diary and retrospective recall assessment

Participants were instructed to fill out a prospective headache diary in paper format with daily entries throughout the baseline period and treatment period. Moreover, participants were asked at each scheduled visit to retrospectively estimate the number of days with headache, migraine, aura, and acute medication use within the preceding month. Details of the headache diary are presented in Supplementary Appendix 6.

#### Patient-Reported Outcomes (PROs)

Participants were instructed to complete nine patient-reported outcomes at Screening, Day 1 (1^st^ dose), and every 12^th^ week throughout the treatment period and the subsequent follow-up period without treatment. All PROs were administered using the online Research Electronic Data Capture (REDCap) software. The complete list of PROs is provided in Supplementary Appendix 7.

#### Adverse events and medications

At all study visits, we recorded the use of concomitant medications and any adverse events (AEs) or serious adverse events (SAEs), which were graded according to the Common Terminology Criteria for Adverse Events (CTCAE) version 4.0 [17].

### Biochemistry core

Participants in the Biochemistry Core had blood samples collected at three time points: Screening (Weeks -6 to -5), Week 24 (± 2 weeks), and Week 48 (± 4 weeks). For participants who were also part of the MRI Core or Provocation Core, blood samples were collected on the day of their first MRI scan or the day of the provocation study with infusion of CGRP. Healthy controls had blood samples collected at one scheduled site visit.

#### Collection

Blood was obtained by antecubital phlebotomy and collected into different tubes: serum gel-separator clot-activator tubes (16 mL), dipotassium ethylenediaminetetraacetic acid (K2 EDTA) tubes (22 mL) and lithium heparin tubes (26 mL). Two of the lithium heparin-tubes (18 mL) contained 2.500 kIE aprotinin (Trasylol®, Nordic Drugs, Denmark) and were pre-cooled before the sampling. A subset of participants had a pre-cooled BD™ P100 blood collection tube (8.5 mL) drawn at Screening (Weeks -6 to -5) and Week 24 (± 2 weeks). Before phlebotomy, the following information was recorded: the presence of a headache, headache characteristics, accompanying symptoms, days since the last migraine attack, aura, menstruation, and the use of acute medications within the last 72 h.

#### Processing and storage

After collection, serum tubes were stored at room temperature for 30 min before centrifugation for 10 min. The remaining blood samples were immediately centrifuged, aliquoted, and stored at –80 °C. Coded labelling and random arrangement of samples were used to ensure that the assays were conducted without knowledge of group assignment.

#### Analyses

Pre-planned analyses included adrenomedullin, amylin, CGRP, pituitary adenylate cyclase activating polypeptide-38 (PACAP-38), vasoactive intestinal polypeptide (VIP), tumor necrosis factor-α (TNF-α), interleukin (IL)-1β, IL-6, IL-10, soluble urokinase plasminogen activator receptor (suPAR), high-sensitivity C-reactive protein (hs-CRP), neurofilament light chain (NfL), glial fibrillary acidic protein (GFAP), estradiol, progesterone, and prolactin.

### MRI Core

Participants in the MRI Core underwent scans at three time points: Screening (Weeks -6 to -5), Week 24 (± 2 weeks), and Week 48 (± 4 weeks). Healthy controls underwent a single scan session.

#### Acquisition

Cerebral imaging was conducted using a 3-Tesla MRI scanner (Siemens MAGNETOM Prisma, Germany) with a 32-channel head coil and without the use of any contrast agents. The total scan time was 55 min. To minimize motion artefacts, foam pads were placed by both temporal regions. Participants were instructed not to take analgesics, antiemetics, antihistamines, benzodiazepines, or anti-inflammatory medications for 48 h prior to the scan session. In addition, they were instructed not consume alcohol, caffeinated foods, or beverages within 12 h of the scanning session. Before the scans, information was collected regarding headache and use of acute medications, as described above for the Biochemistry Core. The sequences selected to identify structural, functional, and neuroinflammatory cerebral changes are presented in Supplementary Appendix 8. Details of pre- and post-processing will be reported in future publications.

### Provocation core

#### CGRP-Provocation

The Provocation Core study involved an open-label, single-arm experiment in which participants received continuous intravenous infusion of CGRP (Tocris Bioscience, United Kingdom) over 20 min (1.5 µg/min). Participants arrived non-fasting between 08:00 AM and 11:00 AM, and had to be free from headache and without intake of analgesics and other acute medications for migraine for 24 h before CGRP infusion. Participants were placed in a supine position, and a catheter was inserted into the antecubital vein to administer the drug using a time- and volume-controlled infusion pump. Vital signs, headache features, use of rescue medication, and AEs were monitored every 10^th^ minute, from 10 min before start of the infusion until discharge 60 min after infusion start. After discharge, participants were asked to complete a headache diary in paper form with hourly entries until 12 h after the infusion start.

### Outcome measures

The outcomes measures are presented below for each REFORM Core. Primary, secondary, and exploratory outcomes will be reported in future publications.

#### Clinical core

- The following data recorded prospectively in a daily headache diary from the baseline period to the final visit: MMDs, monthly headache days (MHDs), monthly moderate-to-severe headache days, monthly days with aura, monthly days with menstruation, and monthly use of acute medication. A migraine day was defined as a day with self-reported migraine, aura and headache, or use of acute migraine-specific medication (triptans, ergotamine derivates, ditans, or gepants). In future publications, we will define the primary efficacy outcome as the proportion of participants achieving a ≥ 50% reduction in mean MMDs, comparing the baseline period to weeks 13 through 24. Participants who withdrew from the study or were lost to follow-up during the 24-week treatment period will be categorized as non-responders.
- Sociodemographic and clinical data collected during the semi-structured interview. Patient-reported data served as the primary source of information concerning the presence of symptoms and use of concomitant medications. Somatic and psychiatric comorbidities were based on participants’ self-report of a physician diagnosis. In cases where accessible, medical health records were used to validate or enhance the provided anamnestic information.
- PROs were collected at Screening, Day 1 (1^st^ dose), Week 12, Week 24, Week 36, and Week 48.
- All AEs and SAEs were documented from first dose to the final study visit for all participants who received at least one dose of erenumab.

#### Biochemistry core

- The concentration of candidate blood-biomarkers was measured at Screening, Week 24 (± 2 weeks), and Week 48 (± 4 weeks).

#### MRI Core

- Changes in structural, functional, and neuroinflammatory MRI outcomes were assessed at Screening, Week 24 (± 2 weeks), and Week 48 (± 4 weeks).

#### Provocation core

- The incidence of CGRP-induced migraine attacks and aura was assessed during the 12-h observational period after the start of infusion.

### Statistical analysis

The sample size was determined by the number of participants planned to receive 24 weeks of erenumab as part of the separate, phase IV study (NCT04265755). For the Clinical Core, we aimed to enroll ≥ 700 participants. For the Biochemistry Core, the target enrollment for the first collection of blood samples was ≥ 600 participants, of which 600 had additional blood samples collected at Week 24, and 200 at Week 48. We aimed to collect BD™ P100 blood collection tubes in 100 participants at Screening and 50 at Week 24. For the MRI Core, we aimed to enroll ≥ 250 participants for the first MRI session. Among those who completed the first MRI session, we considered it feasible to have 100 participants complete both MRI sessions at Week 24 and at Week 48. The Provocation Core was estimated to include 400 participants, with a predefined interim analysis after the completion of 100 provocation studies, to determine whether further enrollment was meaningful. For healthy controls, we aimed to enrolled ≥ 150 individuals.

Detailed statistical methods, including identification of predictors and selection of variables, will be included in future publications. For this article, results were presented descriptively using mean ± standard deviation (SD), or median and interquartile range (IQR) for continuous variables, as appropriate. Categorical data were presented with frequency and percentage. We used complete case analysis for baseline data and presented all instances of missing data. Statistical analyses for this article were performed using R (version 4.1.1) [18].

## Results

**Fig. 2:**
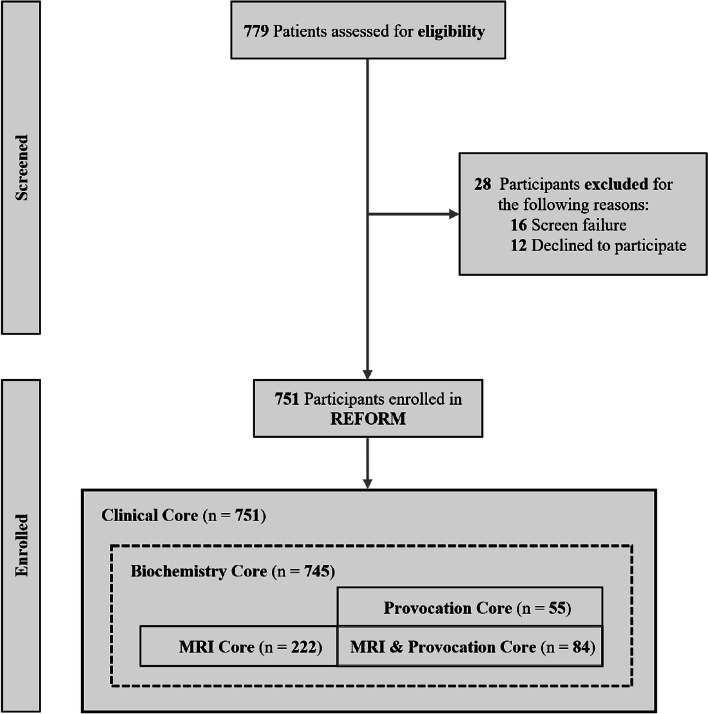
Study flow diagram

From September 2020 to June 2022, a total of 751 participants with migraine were enrolled in the REFORM study. The flow of participants with migraine and their involvement in each REFORM Core is shown in Fig. 2. For the control group, 160 participants were initially included. However, five were excluded due to incidental findings on brain MRI during the screening period, resulting in the inclusion of a total of 155 healthy controls.

### Demographics and clinical characteristics

Table 1 presents the demographics and baseline characteristics of the study population. A total of 751 participants with a mean age of 43.8 ± 12.2 years were included, with 88.8% (*n* = 667) of them being female and 98.4% (*n* = 739) being White. The mean body mass index was 25.1 ± 5.1 kg/m^2^, and the mean age at onset of the disease was 19.3 ± 10.3 years. At the screening visit, the mean MMDs were 14.5 ± 7.0 days, and 64.7% (*n* = 486) of participants were diagnosed with chronic migraine. Furthermore, 30.2% (*n* = 227) of participants experienced migraine aura.

**Table 1:** Demographics and baseline clinical characteristics of the study population

**Demographic characteristics**	**Total (*N*** **= 751)**
Age, mean ± SD [range], years	43.8 ± 12.2 [18–75]
Female sex, n (%)	667 (88.8%)
Racial identity, White, n (%)	739 (98.4%)
Weight, mean ± SD, kg	72.7 ± 16.2
Height, mean ± SD, cm	169.9 ± 7.5
Body mass index, mean ± SD, kg/m^2^	25.1 ± 5.1
Full- or part-time employment or studies, n (%)	562 (74.8%)
**Clinical characteristics**	
Chronic migraine, n (%)	486 (64.7%)
History of aura, n (%)	227 (30.2%)
Medication-overuse headache, n (%)	266 (35.4%)
Age at onset of migraine, mean ± SD, years ^a^	19.3 ± 10.3
Disease duration, mean ± SD, years ^a^	24.4 ± 13.2
Headache frequency (30 days before screening), mean ± SD	
Monthly migraine days (MMDs)	14.5 ± 7.0
Monthly headache days (MHDs)	19.7 ± 7.8
Monthly days with acute medication use ^b^	12.1 ± 6.6
Somatic comorbidity, ongoing, n (%)	
Neck pain	328 (43.7%)
Allergies including hay fever	256 (34.1%)
Lower back pain	224 (29.8%)
Constipation	141 (18.8%)
Tinnitus	128 (17.0%)
Autoimmune conditions	90 (12.0%)
Asthma	87 (11.6%)
Psychiatric comorbidity, ongoing, n (%)	
Depression	81 (10.8%)
Anxiety	81 (10.8%)

Comorbidities currently present in ≥ 10% of participants are shown and arranged by frequency

^a^ missing data for 1 participant

^b^ missing data for 2 participants

Of the comorbidities commonly observed in the study population, neck pain was the most prevalent (43.7%; *n* = 328). Allergies, including hay fever, were reported by 34.1% (*n* = 256), while 29.8% (*n* = 224) reported lower back pain. Constipation was present in 18.8% (*n* = 141), and 17.0% (*n* = 128) reported tinnitus. Autoimmune conditions were present in 12.0% (*n* = 90), while 11.6% (*n* = 87) had asthma. Furthermore, the same proportion of participants had ongoing major depressive episodes and anxiety disorders, with 10.8% (*n* = 81) having a current diagnosis of each condition.

### Treatment patterns

Table 2 shows the treatment patterns of the study population. Of 751 participants with migraine, 97.3% (*n* = 731) reported current use of acute headache medication. Triptans were the most commonly used acute medication, with 89.3% (*n* = 671) participants reporting current use. The specific triptans used by the study population are presented in the Supplementary Appendix 9. Non-opioid combination analgesics consisting of acetylsalicylic acid with caffeine were used by 39.7% (*n* = 298), while 38.9% (*n* = 292) used paracetamol, and 35.6% (*n* = 267) used NSAIDs. Only 1.2% (*n* = 9) reported never using triptans before.

**Table 2:** Treatment patterns of the study population

	**Current use (*N* = 751)**		**Prior failure **^**a**^** (*N* = 751)**	
**Acute medications for headache (≥ 1)**	**n**	**%**	**n**	**%**
Any	731	97.3	712	94.8
Triptans ^b^	671	89.3	466	62.1
Combination analgesics without opioids	298	39.7	318	42.3
Paracetamol	292	38.8	423	56.3
NSAIDs	267	35.6	489	65.1
Combination analgesics with opioids	55	7.3	310	41.3
Opioids (non-combination)	29	3.9	178	23.7
Ergot alkaloids	6	0.8	29	3.9
Gepants	1	0.1	0	0
Ditans	0	0	0	0
**Preventive medications for migraine (≥ 1)**	**n**	**%**	**n**	**%**
Any	364	48.5	667	88.8
Anti-hypertensives	217	28.9	621	82.7
Candesartan	139	18.5	478	63.6
β-blockers	70	9.3	460	61.3
Lisinopril	17	2.3	207	27.6
OnabotulinumtoxinA	98	13.0	79	10.5
Anti-convulsants	65	8.7	465	61.9
Topiramate	41	5.5	430	57.3
Lamotrigine	17	2.3	59	7.9
Valproic acid	5	0.7	77	10.3
Anti-depressants	43	5.7	254	33.8
Amitriptyline	36	4.8	210	28.0
Mirtazapin	3	0.4	65	8.7
Venlafaxin	2	0.3	11	1.5
Anti-CGRP monoclonal antibody	0	0	1	0.1
Gepants	0	0	0	0

Acute and preventive medication groups arranged by frequency of current use

Specific migraine preventive medications currently used by ≥ 2 participants are shown

^a^ Failure due to lack of tolerability and/or lack of efficacy, granted that treatment was of at least the minimally effective dose and duration suggested by the European Headache Federation consensus guideline [19]

^b^ Specific triptans used by the study population are presented in Supplementary Appendix 9

Concomitant preventive medications for migraine were used by 48.5% (*n* = 364). The most commonly used preventive medication were candesartan (18.5%; *n* = 139), onabotulinumtoxinA (13.0%; *n* = 98), β-blockers (9.3%; *n* = 70), and anticonvulsants (8.7%; *n *= 65). Nutraceuticals were used by 13.7% (*n* = 103), with magnesium being used by 12.9% (*n* = 97), and riboflavin (B_2_) being used by 3.2% (*n* = 24).

The most commonly failed preventive medications were candesartan (63.6%; *n* = 478), β-blockers (61.3%; *n* = 461), and topiramate (*n* = 430; 57.3%). The median (IQR) number of failed preventive medications was 3 (2–5), excluding nutraceuticals. Due to intolerability and/or lack of efficacy, 39.9% (*n* = 300) had failed ≥ 4 preventive medications, while 48.9% (*n* = 367) had failed 1–3 medications.

Medications targeting CGRP signaling were seldom used by the study population. Only a few participants had a history of treatment with such medications. Specifically, only one participant discontinued fremanezumab due to lack of efficacy, while two participants discontinued fremanezumab and one participant discontinued galcanezumab due to unavailability. In addition, only one participant reported using ubrogepant, with the indication being acute treatment of migraine attacks.

## Discussion

The REFORM study was a single-center, prospective, longitudinal, observational cohort study conducted to identify predictors of response to erenumab and biomarkers for migraine. The study included 751 participants with migraine, of whom 88.8% were female, which is higher than that of the general population [20], but consistent with that of people with migraine in other tertiary care units [21–23]. The study population had a high burden of disease, with 64.7% diagnosed with chronic migraine, 35.4% with MOH, and an average of 14.5 ± 7.0 MMDs per month. Headache frequency was comparable to reports from some tertiary headache clinics [21], while others has reported lower [22, 23]. The high migraine burden might, in part, be due to the inclusion criteria of at least four MMDs in the three months prior to screening.

Migraine is associated with several comorbidities, which contribute to the overall disease burden [24]. In the REFORM study, anxiety and depression were equally prevalent (10.8%) among participants. Both conditions are more prevalent in people with migraine than in the general population, as for other chronic pain disorders, and have been linked to increasing headache frequency [24–27]. Furthermore, several somatic comorbidities were prevalent in our study population, including asthma, allergies, constipation, neck- and back pain, and tinnitus, which have also been associated with migraine [24, 28, 29]. Of note, a recent study in a large cohort of Polish patients with migraine without aura found that 35.9% had a diagnosis of allergies, which is in line with this study (34.1%) [30].

Participants in the REFORM study frequently used triptans (89.3%) and preventive medications (48.5%). Compared to other specialized headache clinics, they used candesartan more frequently (18.5%), while the use of onabotulinumtoxinA was less common (13.0%) [21]. The restricted use of onabotulinumtoxinA is likely attributed to local practice guidelines, requiring patients to meet specific criteria before treatment commencement. These criteria include a diagnosis of chronic migraine without MOH and a documented lack of response to at least one antihypertensive and one anticonvulsant medication used for migraine prevention. Moreover, the broader use of onabotulinumtoxinA might also have been restricted due to limited staff availability, particularly during the peak of the COVID-19 pandemic. Topiramate was currently used by only 5.5% of participants, while 57.3% had previously failed treatments with this medication. Corresponding numbers for amitriptyline were 4.8% and 28.0%, respectively. High discontinuation rates of these medication might be due to their limited efficacy and poor tolerability [31]. A large proportion of participants (39.9%) had failed four or more preventive medications. It should be noted that previous studies have suggested that a higher number of previous treatment failures may predict lower efficacy of mAbs against CGRP signaling [32–34]. Conversely, the open-label administration of erenumab may inflate treatment responses [35].

The REFORM study's clinical deep phenotyping allows for exploration of clinical predictors for efficacy of erenumab, as well as treatment-induced biomarker changes and patterns in migraine and its subtypes. Previous studies have proposed several positive predictors of treatment response to mAbs against CGRP signaling, including unilateral pain localization [32, 36, 37], response to triptans [32, 38, 39], shorter duration of MOH [32, 40, 41], and older age [42]. In contrast, poor response has been associated with anxiety disorders and a higher number of failed preventive medications [34, 40, 43]. However, previous studies have reported conflicting findings regarding the predictive value of baseline migraine frequency [37, 44], and Migraine Disability Assessment Scale (MIDAS) scores [34, 40, 45].

CGRP is the most studied blood-based biomarker in migraine [7, 46]. The results of studies investigating CGRP levels in patients with episodic migraine and chronic migraine during interictal and ictal states have been inconclusive [46]. Therefore, the value of CGRP for diagnosing migraine or determining disease severity has yet to be established [46]. A few studies have investigated the prediction of treatment efficacy to mAbs against CGRP signaling using blood-based measurements of CGRP [7, 46]. In a recent study of 94 patients with migraine treated with erenumab, baseline serum CGRP levels were not found to predict treatment response, but the treatment period was only 2–4 weeks [10]. Another recent study found that 96 patients with chronic migraine showed elevated levels of serum CGRP compared to healthy controls, and that these levels subsequently normalized at two weeks and at three months during treatment with erenumab [11]. However, pretreatment serum CGRP levels did not predict response to erenumab [11]. In a study that evaluated salivary CGRP in 24 participants, baseline levels predicted ≥ 50% reduction in response to erenumab in MHDs in episodic but not chronic migraine [9]. However, no significant overall changes in salivary CGRP levels were detected after 12 weeks of treatment with erenumab [9]. Apart from CGRP, the selection of other biomarkers in REFORM study was based on their involvement in the proposed mechanisms of migraine pathophysiology. These include other neuropeptides than CGRP [3, 47, 48], neurogenic inflammation [49], disrupted blood–brain barrier function [50], astrocytic dysfunction [51], the influence of sex-hormones [52], and abnormal prolactin signaling [53].

MRI studies have reported inconsistent differences between individuals with migraine and healthy controls [7]. Structural MRI studies have reported abnormalities such as white matter hyperintensities and volumetric changes in individuals with migraine [54], while fMRI studies have suggested changes in pain-processing and visual networks [55–57]. Reasons for the failure to consistently replicate these findings may include insufficient sample sizes, heterogeneous populations, and differences in analytical approaches [54, 55, 57]. Few fMRI studies have explored the efficacy predictors of mAbs against CGRP signaling, or erenumab-induced biomarker changes. One study of 27 participants treated with erenumab over two weeks found reduced activation in multiple brain areas in uncorrected analyses, and reduced hypothalamic activation in response to painful trigeminal stimulation in responders only [58]. Another recent study of 26 participants treated with galcanezumab found higher baseline activation of the spinal trigeminal nucleus correlated with reduction of MHDs after three months treatment [59]. In a third study of 32 participants receiving erenumab, responders exhibited differences in resting-state functional connectivity and central processing of thermal painful stimuli after eight weeks of treatment [12]. This study also found that after eight weeks of treatment, responders showed significantly less iron deposition in key areas for pain processing, including the periaqueductal gray (PAG) and anterior cingulate cortex [13]. However, limitations included lack of healthy controls, use of abortive migraine medications, and limited sample size [12, 13]. We sought to address these limitations in the REFORM study. In addition, we conducted follow-up MRI scans after 24 weeks of treatment with erenumab to allow more time for neuronal plastic adaptations to occur, and used corrected analysis to reduce false-positive findings [60].

Human provocation studies that use trigger substances, such as CGRP, have provided important insights into the signaling pathways and drug targets that are relevant to migraine [61]. However, only one provocation study of 13 patients has investigated prediction of efficacy of mAbs against CGRP signaling [62]. This study found that participants who reported a good response to erenumab were more susceptible to developing migraine attacks after infusion of CGRP, with a positive predictive value of 0.80. However, due to the low number of participants with a poor response, no firm conclusions could be drawn regarding whether the absence of a migraine attack predicted a poor response [62]. In the REFORM study, we used a larger sample size, and assessed efficacy prospectively, instead of retrospectively [62].

Recent studies have examined the impact of discontinuing monoclonal antibodies (mAbs) against CGRP signaling on the evolution of headache and migraine frequency [63]. A study including 62 patients with episodic and chronic migraine treated with erenumab, galcanezumab, or fremanezumab found that approximately three months after treatment cessation, migraine frequency returned to baseline levels [63]. Although some studies have shown comparable results following mAb discontinuation [64, 65], others found the impact to be less pronounced [66, 67]. In the follow-up phase of REFORM, we will explore the clinical course, changes in blood-based and MRI biomarkers, and predictors of sustained response after discontinuation.

### Limitations

The REFORM study has certain limitations that need to be addressed. First, convenience sampling was chosen due to feasibility, but can lead to unbalanced selection of participants and result in sampling bias. Second, although paper diaries were used, electronic headache dairies with time stamps are generally preferable to reduce recall bias, missing data, and errors in data completion [68]. Third, the population was heterogenous in several respects. These included differences in the use of preventive medications, which were not paused for ethical reasons, and that blood samples and MRI were not acquired under specific ictal or interictal conditions due to high frequency of migraine attacks in the study population. While the latter provides an opportunity to examine the ictal phase of migraine in a large sample, other subgroups will also likely be of sufficient size due to the large study population. Furthermore, statistical adjustments can be made to address heterogeneity by controlling for potential covariates. Fourth, the sample population reflected that of tertiary headache clinics, and therefore direct extrapolation of results to individuals with migraine in the general population may not be possible. However, investigating a population with a more severe migraine phenotype may increase the likelihood of detecting biomarker differences characteristic of migraine. Finally, the results may also not be fully generalizable to some racial or ethnic minority groups, as most participants were female and White.

## Conclusions

The REFORM study is a single-center, prospective, observational study designed to identify predictive biomarkers for the efficacy of erenumab. A total of 751 participants with migraine were enrolled in the study, all of which were scheduled for 24 weeks of preventive treatment with erenumab, and 24 weeks of follow-up after discontinuation. The sociodemographic and clinical characteristics of the participants showed a high burden of migraine and frequent use of acute and preventive medications, which is representative of populations in tertiary headache clinics. The details of the methodology and the results of the Clinical, Biochemistry, MRI, and Provocation Cores presented in this article will be published in future publications.

## Supplementary Information

**Additional file 1: Supplementary Appendix 1.** Inclusion and Exclusion Criteria.** Supplementary Appendix 2.** Inclusion and Exclusion Criteria.** Supplementary Appendix 3.** Inclusion and Exclusion Criteria.** Supplementary Appendix 4.** Study Instruments and Assessed Domains.** Supplementary Appendix 5.** Semi-Structured Interview.** Supplementary Appendix 6.** Headache Diary Details.** Supplementary Appendix 7.** Patient-Reported Outcomes.** Supplementary Appendix 8.** MRI Sequences.** Supplementary Appendix 9.** Use of Triptans in the Study Population.

## Data Availability

The data used and/or analyzed during the current study are available from the corresponding author on reasonable request.
