# Supplementary material for: The Registry for Migraine (REFORM) study: methodology, demographics, and baseline clinical characteristics
Source: J Headache Pain. 2023 Jun 12;24(1):70. doi: 10.1186/s10194-023-01604-2 (PMC10257973; doi:10.1186/s10194-023-01604-2)
Supplement: Supplementary file 1 — Additional file 1: Supplementary Appendix 1. Inclusion and Exclusion Criteria. Supplementary Appendix 2. Inclusion and Exclusion Criteria. Supplementary Appendix 3. Inclusion and Exclusion Criteria. Supplementary Appendix 4. Study Instruments and Assessed Domains. Supplementary Appendix 5. Semi-Structured Interview. Supplementary Appendix 6. Headache Diary Details. Supplementary Appendix 7. Patient-Reported Outcomes. Supplementary Appendix 8. MRI Sequences. Supplementary Appendix 9. Use of Triptans in the Study Population. [file 10194_2023_1604_MOESM1_ESM.docx]

**Supplementary Appendix 1. Inclusion and Exclusion Criteria**

| **Clinical and Biochemistry Core** |
| --- |
| *Inclusion criteria:*   - ≥18 years of age at screening - History of migraine without aura, migraine with aura, or chronic migraine according to the International Classification of Headache Disorders (ICHD) version 3^rd^ edition criteria (code 1.1, 1.2 or 1.3) for ≥1 year prior to study entry - ≥4 monthly headache days that meet criteria as migraine days on average for three months prior to study screening - Preventive treatment with erenumab scheduled at the Danish Headache Center, Department of Neurology, University Hospital Rigshospitalet - Provision of informed consent prior to initiation of any study-related activities/procedures   *Exclusion criteria:*   - >50 years of age at migraine onset - History of persistent post-traumatic headache, hemiplegic migraine, or cluster headache (ICHD-3 code 5.2, 1.2.3, and 3.1) - Inability to differentiate migraine headache from other headaches - Risk of self-harm or harm to others as evidenced by past suicidal behavior - History or evidence of any other clinically significant disorder, condition, or disease (except those outlined above), that might pose a risk to subject safety or interfere with study evaluation - Previous treatment with erenumab - Treatment with another anti-CGRP monoclonal antibody for three months prior to screening - Concomitant preventive medication apart from anti-CGRP antibodies was allowed, but only if dosage was stable for two months prior to the screening - Female participants of childbearing potential unwilling to use acceptable methods of contraception (oral contraceptives, intrauterine device, intrauterine hormonal-releasing system, bilateral tubal ligation/occlusion, vasectomized partner, sexual abstinence, male or female condom, cap, diaphragm, or sponge with spermicide) - Known sensitivity to any products or components to be administered - Not likely to be able to complete all protocol required study visits or procedures, and/or comply with all required study procedures to the best of the subject and study investigator’s knowledge |

**Abbreviations**: CGRP, calcitonin gene-related peptide; ICHD-3: International Classification of Headache Disorders, 3^rd^ edition.

**Supplementary Appendix 2. Inclusion and Exclusion Criteria**

| **MRI Core** |
| --- |
| *Same as for Clinical and Biochemistry Core (see Supplementary appendix 1), along with the following* e*xclusion criteria:*   - Magnetic implants or foreign objects (e.g., insulin pumps or pacemakers) - Surgical procedures within six weeks prior to scan - Severe claustrophobia - Previous use of sedatives for MRI - Prior pathological findings on MRI or CT of the brain - Unwillingness to receive information regarding incidental findings |
| **Provocation Core** |
| *Same as for Clinical and Biochemistry Core (see Supplementary appendix 1), along with the following* e*xclusion criteria:*   - Incessant headache - Hypertension on the experimental day defined as systolic blood pressure ≥150 mmHg or diastolic blood pressure ≥100 mmHg, or hypotension on the experimental day defined as systolic blood pressure <90 mmHg or diastolic blood pressure <50 mmHg - Participants were not allowed to have headache or consume analgesics or migraine-specific medications within 24 hours of CGRP infusion |

**Abbreviations**: CGRP, calcitonin gene-related peptide; CT, computed tomography; MRI, magnetic resonance imaging.

**Supplementary Appendix 3. Inclusion and Exclusion Criteria**

| **Healthy Volunteers** |
| --- |
| *Inclusion criteria:*   - ≥18 years of age at screening   *Exclusion criteria:*   - History of primary headache disorders (except infrequent episodic tension-type headache) - History of secondary headache disorders - Any first-degree relative with a history of primary headache disorders (except ≤5 monthly days with tension-type headache) - Headache within 24 hours of any study-related task or procedure - Past suicidal behavior or risk of self-harm - Daily use of medications except oral contraceptives - History or evidence of any other clinically significant disorder, condition, or disease that, in the opinion of the site investigator, would pose a risk to participant safety or interfere with study evaluation, procedures or completion - History or evidence of any psychiatric disorder - Female study participants of childbearing potential with a positive pregnancy test during the study visit - Pregnancy or breastfeeding, female study participants unwilling to use one acceptable method of effective contraception (oral contraceptives, intrauterine device, intrauterine hormonal-releasing system, bilateral tubal ligation/occlusion, vasectomized partner, sexual abstinence, male or female condom, cap, diaphragm, or sponge with spermicide) - Contraindications to magnetic resonance imaging (MRI) - Unlikely to comply with and complete all protocol-required procedures to the best of the study participant and the study investigator’s knowledge. |

**Abbreviations**: MRI, magnetic resonance imaging.

**Supplementary Appendix 4. Study Instruments and Assessed Domains**

| **Domains** | **Instruments** |
| --- | --- |
| Sociodemographics and medical history | - Semi-structured interview |
| Headache phenotype | - Semi-structured interview - Headache diary (baseline period) |
| Headache frequency | - Semi-structured interview - Headache diary - Retrospective recall assessment |
| Headache disability and non-cephalic pain | - PROs (ASC-12, CSI, HIT-6, NDI, ODI) |
| Acute migraine treatment | - Semi-structured interview - Headache diary - Retrospective recall assessment |
| Preventive migraine treatment | - Semi-structured interview - RedCAP Online survey |
| Cognitive function | - PROs (HIT-6, WHODAS) |
| Vitality and social function | - PROs (HIT-6, MIDAS, WHODAS) |
| Psychological distress | - PROs (HADS, HIT-6) |
| Sleep | - PROs (PSQI) |

**Abbreviations**: ASC-12, 12-item Allodynia Symptom Checklist; CSI, Central Sensitization Index; HADS, Hospital Anxiety and Depression Scale; HIT-6, Headache Impact Test-6; MIDAS, Migraine Disability Assessment questionnaire; NDI, Neck Disability Index; ODI, Oswestry Low Back Pain Disability Index; PROs, patient-reported outcomes; PSQI, Pittsburgh Sleep Quality Index; RedCAP, Research Electronic Data Capture; WHODAS 2.0, World Health Organization Disability Assessment Schedule 2.0.

**Supplementary Appendix 5. Semi-Structured Interview**

| **Domains** | **Items** |
| --- | --- |
| Basic information | - Age, sex, and racial identity - Height, weight, and handedness |
| Sociodemographics | - Marital status and no. of children - Occupational status and estimated monthly income before taxes - Highest achieved education, including no. of years - Family history of migraine |
| Migraine | - Type (without aura, with aura, or both) and age of onset - Retrospectively estimated migraine days within last month, last three months, last year, and lifetime - Headache duration, location, characteristics, and intensity - Accompanying symptoms, including most bothersome - Autonomic symptoms - Aura (visual, tactile, speech, motor, brainstem, retinal) - Provoking factors - Premonitory and postdromal symptoms |
| Tension-type headache | - Age of onset - Headache duration, location, characteristics, and intensity - Accompanying symptoms - Provoking factors - Premonitory symptoms |
| Menstruation | - Age at menarche, and menopause if relevant - Menstrual cycle duration, regularity, and association with migraine |
| Acute and preventive medications | - Type(s) of medications currently used and previously failed^*^ - Average monthly no. of days with use of acute medication by type within the last three month |
| Non-pharmacological treatment | - Type(s) of treatments currently used and previously failed |
| Vestibular migraine & Visual snow | - Descriptions of symptoms |
| Comorbidities | - Presence and history of symptoms - Medical records, and self-report of physician diagnosis |
| Active headache diagnoses | - Headache diagnoses according to ICHD-3 within last 12 months |

**Abbreviations:** ICHD-3, the International Classification of Headache Disorders, 3^rd^ edition.

*: Failure of previous migraine preventive therapies were defined as lack of tolerability and/or lack of efficacy, granted that treatment was of at least the minimally effective doses and durations proposed by the European Headache Federation consensus guideline [19].

**Supplementary Appendix 6. Headache Diary Details**

| **Study period** | **Items** |
| --- | --- |
| Full study (baseline to week 48) | - Presence and intensity of headache rated as 0 (no pain); 1 (mild pain); 2 (moderate pain); and 3 (severe pain) [16]. - Migraine based on assessment of the participant - Presence of aura - Menstruation - Use and type of acute medication for headache or migraine |
| Additional data captured during baseline period only | - Headache duration (hours) - Headache characteristics   - Unilateral localization   - Pulsating quality   - Exacerbation by routine physical activity - Associated symptoms   - Photophobia   - Phonophobia   - Nausea   - Vomiting - Aura type:   - Visual aura   - Sensory aura   - Aura involving speech and/or language |

**Supplementary Appendix 7. Patient-Reported Outcomes (PROs)**

| **Instrument** | **Abbreviation** | **Description** |
| --- | --- | --- |
| 12‐item Allodynia Symptom Checklist | ASC‐12 | Twelve-item self-reported questionnaire used to assess the prevalence and severity of allodynia in a migraine population. |
| Central Sensitization Index | CSI | Thirty-five-item self-reported questionnaire designed to identify patients with symptoms that may be related to central sensitization or central sensitivity syndromes such as fibromyalgia, neck injury, temporomandibular joint disorder, and/or migraine/tension-type headache. |
| Hospital Anxiety and Depression Scale | HADS | Seven-item self-reported questionnaire used to measure clinically significant anxiety and depression in a general medical population. |
| Headache Impact Test-6 | HIT-6 | Six-item self-reported questionnaire used to monitor the impact headaches have on patients’ cognitive-, social and role functioning, psychological distress, and vitality. |
| Migraine Disability Assessment questionnaire | MIDAS | Five-item self-reported questionnaire which captures information on time lost from work, housework or chores, and leisure activities. |
| Neck disability index | NDI | Ten-item self-reported questionnaire used to determine how neck pain affects a patients’ daily life and to assess the self-rated disability of patients with neck pain. |
| Oswestry Low Back Pain Disability Index | ODI | Ten-item self-reported questionnaire used to measure the level of functional disability for patients with low back pain. |
| Pittsburgh Sleep Quality Index | PSQI | Nine-item self-reported questionnaire used to measure sleep quality and patterns in adults. It measures seven components of sleep: subjective sleep quality, sleep latency, sleep duration, habitual sleep efficiency, sleep disturbances, use of sleeping medications, and daytime dysfunction over the last month. |
| WHO Disability Assessment Schedule 2.0 | WHODAS 2.0 | Twelve-item self-reported questionnaire used to measure generic health and disability. It is used across all diseases including mental, neurologic, and addictive disorders. It covers six domains of functioning including cognition, mobility, self-care, social interactions, life activities, and participation in community activities. |

**Supplementary Appendix 8. MRI Sequences**

| **Structural MRI**   - Magnetization-prepared rapid acquisition gradient echo (MPRAGE) - Magnetization-prepared 2 rapid acquisition gradient echo (MP2RAGE) - Combined generalized auto-calibrating partially parallel acquisition and model-based accelerated relaxometry by iterative nonlinear inversion (GRAPPATINI) - Fluid-attenuated inversion recovery (FLAIR) - Diffusion tensor imaging (DTI) - Gradient recalled echo (GRE) with and without magnetization transfer ratio (MTR)   **Functional MRI**   - Resting-state functional MRI (fMRI) - Task-based functional MRI with visual stimulation. |
| --- |

**Supplementary Appendix 9. Use of Triptans in the Study Population**

|  | **Current use (n = 751)** | | **Prior failure* (n = 751)** | |
| --- | --- | --- | --- | --- |
| **Triptans used (≥1 type)** | **n** | **%** | **n** | **%** |
| Any type of triptan | 671 | 89.3 | 466 | 62.1 |
| Sumatriptan | 364 | 48.5 | 323 | 43.0 |
| Rizatriptan | 196 | 26.1 | 204 | 27.2 |
| Eletriptan | 173 | 23.0 | 122 | 16.2 |
| Zolmitriptan | 52 | 6.9 | 90 | 12.0 |
| Naratriptan | 11 | 1.5 | 16 | 2.1 |
| Frovatriptan | 8 | 1.1 | 31 | 4.1 |
| Almotriptan | 8 | 1.1 | 29 | 3.9 |
| Triptans arranged by frequency of current use.  *: Failure due to lack of tolerability and/or lack of efficacy, granted that treatment was of at least the minimally effective dose and duration suggested by the European Headache Federation consensus guideline [19]. | | | | |
